# Long-term cardiovascular function and cardiopulmonary performance in athletes after COVID-19: results from the COSMO study

**DOI:** 10.3389/fcvm.2026.1776089

**Published:** 2026-04-29

**Authors:** Jana Schellenberg, Lynn Matits, Johannes Kersten, Daniel A. Bizjak, Florian Horn, Johannes Hell, Sebastian Viktor Waldemar Schulz, Eric Schwarz, Johannes Kirsten

**Affiliations:** 1 Sports and Rehabilitation Medicine, University Ulm Hospital, Ulm, Germany; 2 Clinical & Biological Psychology, Institute of Psychology and Education, Ulm University Hospital, Ulm, Germany; 3 Herzplus Ulm, Ulm, Germany

**Keywords:** athletes, cardiopulmonary exercise testing (CPET), cardiorespiratory fitness, global longitudinal strain, longitudinal follow-up, mild COVID-19

## Abstract

**Introduction:**

Most studies in athletes have focused on short-term outcomes after SARS-CoV-2 infection, whereas long-term data on cardiac function and exercise performance remain limited. This prospective study evaluated 12-month changes in cardiac structure, myocardial deformation, and cardiopulmonary performance in elite and recreational athletes after mild COVID-19.

**Methods:**

Fifty-two athletes (median age 32.5 (24.8–46.3) years; 27 females) from endurance, power, and mixed sports underwent echocardiography, cardiopulmonary exercise testing (CPET), and bioimpedance analysis after infection (T0; median 88 days post-infection) and again at 12-month follow-up (T1). Measurements included left and right ventricular global longitudinal strain (LV/RV GLS), RV free wall strain (FWS), left atrial strain, CPET performance and ventilatory parameters, and anthropometric variables. Longitudinal changes were evaluated using paired comparisons and linear mixed-effects models. Univariable and multivariable regressions were performed to identify predictors of follow-up VO_2peak_ and its longitudinal change.

**Results:**

Cardiac structure, LV and RV systolic function, and myocardial deformation remained stable over 12 months, with no evidence of adverse remodeling. A significant reduction in E/E′ medial was observed at follow-up (*p* = 0.008), while all other diastolic indices did not change significantly. Cardiopulmonary performance was preserved, with no significant changes in maximal workload, absolute or relative VO_2peak_, ventilatory efficiency, or oxygen pulse (all *p* > 0.05). CPET differences were limited to a higher peak RER (*p* = 0.045) and a lower resting heart rate at T1 (*p* = 0.010). Body composition changed modestly, with higher BMI (*p* = 0.035), while pulmonary function parameters remained stable. In multivariable linear regression, baseline VO_₂peak_ was the only independent predictor of follow-up VO_₂peak_ (*β* = 0.92, 95% CI 0.68–1.15; *p* < 0.001; R² = 0.64), whereas sex, sport type, and baseline LV GLS were not associated with performance at T1.

**Conclusion:**

Athletes recovering from mild SARS-CoV-2 infection show no evidence of impaired myocardial structure, strain, or cardiopulmonary performance at 12 months post-infection. Minor variations in diastolic indices and body composition appeared physiological, and baseline aerobic fitness was the strongest predictor of follow-up performance. These findings indicate a stable 1-year cardiopulmonary outcome and support the safety of return to sport in asymptomatic athletes.

## 1 Introduction

Severe Acute Respiratory Syndrome-Coronavirus-2 (SARS-CoV-2) infection is primarily a respiratory syndrome characterized by acute lung injury and, in severe cases, respiratory failure (1), but it has also been associated with a broad spectrum of cardiovascular manifestations, including myocardial injury, impaired ventricular function (2), and autonomic imbalance (3). These concerns are particularly relevant in athletic populations, where even subtle alterations in cardiac performance may influence training capacity, competitive readiness, and return-to-play decision-making (4). Early reports suggested possible myocardial involvement after Coronavirus Disease 2019 (COVID-19), including elevations in cardiac biomarkers, abnormalities in echocardiographic deformation indices, and cardiac MRI findings of inflammation or edema (5, 6). However, most of these observations were derived from hospitalized or symptomatic individuals rather than athletes, who typically experience mild disease (7, 8). Clinically significant myocardial involvement in athletes is uncommon, with myocarditis reported in only 1%–3% of screened competitive athletes (9, 10).

Strain imaging—including left and right ventricular global longitudinal strain (LV/RV GLS)—is a sensitive tool for detecting early myocardial dysfunction (11). Impaired LV/RV GLS have been described in hospitalized patients and individuals recovering from COVID-19 across a range of fitness levels (5, 12–15). While some studies indicated persistent reductions in GLS after infection (16, 17), others have demonstrated normalization improvement over time (12, 18, 19), suggesting heterogeneous and incompletely understood recovery patterns. In athletic populations, the clinical relevance of potential subclinical myocardial changes remains uncertain. Most studies in athletes have focused on short-term outcomes within weeks or early months after infection (20–22), and long-term data on cardiac function and exercise performance are limited. Consequently, it remains unclear whether mild SARS-CoV-2 infection leads to persistent alterations in myocardial deformation, diastolic function, or cardiopulmonary fitness, and whether early echocardiographic findings predict later performance.

This prospective cohort study aimed to evaluate 12-month changes in cardiac structure, myocardial deformation, and cardiopulmonary performance in elite and recreational athletes following mild SARS-CoV-2 infection. Additionally, we sought to identify predictors of long-term exercise capacity and to assess whether longitudinal changes in cardiac function or body composition were associated with performance changes. By integrating advanced echocardiography, cardiopulmonary exercise testing (CPET), and anthropometric analysis, this study provides a comprehensive evaluation of long-term cardiovascular recovery in athletes after mild COVID-19.

## 2 Materials and methods

### 2.1 Study population

Elite and recreational athletes participating in the COVID-19 in Elite Sports—a Multicenter Cohort Study (COSMO-S) at the Department of Sports and Rehabilitation Medicine, University of Ulm, underwent a baseline assessment after a first SARS-CoV-2 infection and a follow-up examination twelve months later (23). Inclusion criteria were age ≥18 years, confirmed SARS-CoV-2 infection by PCR or serology with typical symptoms, and classification as recreational or elite athletes, defined as participation in endurance, power, mixed or technical sports (24) with a training frequency of ≥3 sessions per week (>20 metabolic equivalents of task (MET) per week). Exclusion criteria comprised acute or chronic illnesses preventing physical evaluation, acute SARS-CoV-2 infection at the time of testing, refusal of venous blood sampling, or withdrawal of consent. All participants provided written informed consent. The study followed the Declaration of Helsinki and was approved by the local ethics committee of the University of Ulm (EK 408/20).

### 2.2 Clinical evaluation

All participants underwent a standardized clinical evaluation including medical history, physical examination, venous blood sampling, bioimpedance analyses, a 12-lead electrocardiogram (ECG) and two-dimensional (2D) transthoracic echocardiography (TTE), and cardiopulmonary exercise testing (CPET).

### 2.3 Echocardiography and strain analysis

TTE was performed using an EPIQ 7 ultrasound system with a phased-array X5-1 probe (Philips GmbH, Hamburg, Germany) in accordance with current guidelines (25). LV end-diastolic volume (LVEDV), end-systolic volume (LVESV), ejection fraction (LVEF), and stroke volume (SV) were quantified using the biplane Simpson method. LV internal diameter in diastole and systole (LVIDd/s), interventricular septal thickness (IVSd/s), and posterior wall thickness (PWd/s) were measured in parasternal long-axis views. LV mass was calculated using the guideline-recommended linear method (25). Right ventricular systolic function was assessed by tricuspid annular plane systolic excursion (TAPSE), obtained from the focused four-chamber view using M-mode. Diastolic function was evaluated using mitral inflow velocities (E/A ratio) and the mean E/È ratio derived from septal and lateral mitral annular velocities (26).

Speckle-tracking strain analysis was performed using automated software (AutoStrain, TomTec Imaging Systems, Germany). LV GLS was obtained from apical two-, three-, and four-chamber views, with manual adjustments performed to ensure optimal tracking. RV GLS and RV free-wall strain (RV FWS) were measured from the dedicated RV focused apical four-chamber view. Left atrial strain (LAS) was assessed in the apical four-chamber view and reported as reservoir (LASr), conduit (LAScd), and contraction strain (LASct). Reference values from current consensus documents are provided for contextual interpretation of GLS measurements. However, in this study, GLS was analyzed as a continuous variable, and no categorical classification into normal or pathological values was performed (27–29).

### 2.4 Cardiopulmonary exercise testing (CPET)

CPET was performed on a cycle ergometer (Excalibur Sport, LODE B.V., Groningen, The Netherlands) using a linear ramp protocol designed to achieve exhaustion within 8–12 min, in accordance with the Clinical Recommendations for Cardiopulmonary Exercise Testing (30). Breath-by-breath gas exchange analysis (Ergostik, Geratherm, Geratal, Germany) was used to determine VO_2peak_ (both in L/min and mL/kg/min), calculated as the mean oxygen uptake during the final 30 s of exercise. A continuous 12-lead ECG (Cardiopart 12 Blue/Blue-P, AMEDTEC Medizintechnik Aue GmbH, Aue, Germany) was recorded throughout the test. All CPETs were evaluated by the same investigator following standard criteria by Wasserman et al. (31). The following variables were measured or derived: maximal workload and workload normalized to body weight, peak respiratory oxygen uptake (VO_2peak_), peak respiratory exchange rate (RER), heart rate at peak respiratory oxygen uptake (HR@VO_2peak_), ventilatory parameters including maximal ventilation and ventilatory reserve, oxygen uptake/work rate slope (VO_2_/WR slope), peak oxygen pulse (VO_2_/HR) and the ventilatory equivalent of carbon dioxide slope (VE/VCO_2_ slope).

### 2.5 Statistical analysis

All statistical analyses were performed using JASP (version 0.95.4) (32) and R (version 4.5.2) (33). Baseline characteristics are reported as median with interquartile range (IQR). Results derived from linear mixed-effects models are presented as estimated means ± standard error (SE) with 95% confidence intervals. Categorical variables are reported as counts and percentages. Distributional assumptions and model diagnostics were assessed using the Shapiro–Wilk test and visual inspection of histograms, Q–Q plots and residual plots. Longitudinal changes in echocardiographic, cardiopulmonary, and clinical parameters were primarily evaluated using linear mixed-effects models with Time (T0, T1) as fixed effect and ID as random intercept. This approach accounts for within-subject correlation and allows inclusion of participants with incomplete follow-up without listwise deletion. Associations between continuous variables at baseline (e.g., LV GLS with diastolic parameters, LA strain indices, or VO_₂peak_) were examined using Pearson correlation coefficients. Scatterplots were visually inspected to assess linearity and to identify potential influential outliers. Determinants of LV GLS were examined using univariable and multivariable linear regression models including skeletal muscle mass, age, and sex as independent predictors. Predictors of follow-up exercise capacity were assessed using linear regression models with VO_2peak_ at T1 as the dependent variable and baseline VO_2peak_, sex, and sport discipline as independent variables. In a separate model, baseline LV GLS was entered to evaluate its association with VO_2peak_ at T1. Regression coefficients are reported as unstandardized estimates (b) with 95% confidence intervals. Regression assumptions (linearity, approximate homoscedasticity, and normality of residuals) were evaluated using residual and Q–Q plots. Multicollinearity was examined using variance inflation factors. A *p*-value ≤ 0.05 was considered statistically significant. No correction for multiple testing was applied due to the exploratory nature of the study.

## 3 Results

### 3.1 Cohort characteristics

A total of 52 (27 female/25 male) elite and recreational athletes (median age 32.5 (24.8–46.3) years) were evaluated after their first SARS-CoV-2 infection. The median time between infection and the baseline assessment (T0) was 88 (53–186) days. All participants underwent a follow-up examination approximately twelve months later (T1). Athletes predominantly practiced endurance (*n* = 35), power (*n* = 8), or mixed (*n* = 8) sport disciplines (24) and trained on average six ± four hours per week. All infections were mild; no participant required hospitalization and no cases of myocarditis were detected.

A linear mixed-effects model with Time as fixed effect and ID as random intercept revealed significant changes in BMI [F(1, 51) = 4.689, *p* = 0.035], and resting HR [F(1, 51) = 7.152, *p* = 0.010] from T0 to T1 (Table 1).

**Table 1:** Demographic cohort characteristics.

Variable	Mean ± SE T0 (95% CI)	Mean ± SE T1 (95% CI)	Change T1–T0	*p*-value
BMI (kg/m^2^)	23.67 ± 0.45 (22.80–24.55)	24.06 ± 0.45 (23.18–24.93)	+0.39	**0** **.** 035*
Weight (kg)	72.87 ± 1.94 (69.07–76.68)	73.88 ± 1.94 (70.08–77.68)	+1.01	0.054
BSA (m^2^)	1.88 ± 0.03 (1.82–1.93)	1.86 ± 0.03 (1.83–1.94)	−0.02	0.088
SMM (kg)	32.07 ± 0.96 (30.19–33.94)	32.24 ± 0.96 (30.36–34.11)	+0.17	0.288
Body fat (%)	20.82 ± 1.31 (18.24–23.39)	21.74 ± 1.31 (19.17–24.30)	+0.92	0.054
ECW/TBW ratio	0.38 ± 0.00 (0.38–0.38)	0.38 ± 0.00 (0.38–0.38)	+0.00	0.648
HR (bpm)	67.73 ± 1.55 (64.69–70.77)	63.52 ± 1.55 (60.48–66.56)	−4.21	**0**.010*
SBP (mmHg)	117.7 ± 2.05 (113.7–121.8)	116.9 ± 2.43 (112.1–121.6)	−0.80	0.780
DBP (mmHg)	78.04 ± 1.67 (74.76–81.31)	76.88 ± 1.98 (73.00–80.76)	−1.16	0.648
Creatine kinase (U/L)	129.40 ± 19.69 (90.77–168.00)	189.00 ± 19.69 (150.37–227.60)	+59.60	**0**.005*
Troponin T (ng/L)	5.14 ± 0.46 (4.23–6.05)	5.15 ± 0.99 (3.21–7.10)	+0.01	0.992
CRP (mg/L)	1.14 ± 0.52 (0.12–2.17)	1.71 ± 0.53 (0.67–2.74)	+0.57	0.442

SE, Standard Error; BMI, Body Mass Index; BSA, Body Surface Area; SMM, Skeletal Muscle Mass; ECW/TBW, Extracellular Water/Total Body Water Ratio; HR = Heart Rate at Rest; SBP, Systolic Blood Pressure; DBP, Diastolic Blood Pressure; CRP, C-reactive protein; Significant results are presented as follows: *≤0.05.

### 3.2 Cardiac structure and myocardial deformation

Echocardiographic characteristics at T0 and T1 are presented in Table 2. Left ventricular systolic function remained stable over the period of 12 months, with no significant changes in LVEF, LVEDV, and LV GLS (all *p* > 0.05). LV internal diameters and wall thicknesses demonstrated no significant longitudinal changes (all *p* > 0.05). Diastolic parameters showed a small improvement, with a significant reduction in E/È medial (T0: 7.94 ± 0.25 vs. T1: 7.23 ± 0.25, *p* = 0.008, Cohen`s d = −0.40). The E/A ratio and E/È lateral showed non-significant trends (*p* = 0.058 and *p* = 0.064, respectively). Resting left atrial strain parameters were not associated with E/È medial (reservoir: *r* = –0.08, *p* = 0.437), conduit (*r* = 0.12, *p* = 0.252), and contraction strain (*r* = –0.01, *p* = 0.917).

**Table 2:** Echocardiographic parameters at study time T0 and T1.

Variable	Mean ± SE T0 (95% CI)	Mean ± SE T1 (95% CI)	Change T1–T0	*p*-value
LVEF (%)	73.15 ± 1.20 (70.80–75.50)	73.37 ± 1.21 (71.00–75.74)	0.22	0.897
LVEDV (mL)	124.5 ± 4.63 (115.4–133.60)	121.5 ± 4.63 (112.4–130.60)	−3.00	0.398
LVESV (mL)	33.91 ± 1.93 (30.14–37.69)	32.64 ± 1.93 (28.86–36.41)	−1.27	0.584
LVIDd (mm)	49.89 ± 0.63 (48.66–51.12)	49.18 ± 0.63 (47.94–50.41)	−0.71	0.174
LVIDs (mm)	31.54 ± 0.64 (30.28–32.79)	31.20 ± 0.64 (29.94–32.47)	−0.34	0.625
IVSd (mm)	8.44 ± 0.20 (8.05–8.83)	8.63 ± 0.20 (8.24–9.03)	0.19	0.356
IVSs (mm)	12.47 ± 0.35 (11.78–13.15)	12.33 ± 0.39 (11.56–13.09)	−0.14	0.777
LVPWd (mm)	8.46 ± 0.20 (8.08–8.85)	8.74 ± 0.20 (8.36–9.13)	0.28	0.228
LVPWs (mm)	14.08 ± 0.53 (13.04–15.13)	13.14 ± 0.60 (11.96–14.32)	−0.94	0.228
LV mass (g)	150.5 ± 6.18 (138.4–162.6)	154.1 ± 6.48 (141.4–166.8)	3.60	0.443
SV (mL)	93.16 ± 3.96 (85.41–100.91)	91.00 ± 3.96 (83.25–98.76)	−2.16	0.605
E/A	1.38 ± 0.06 (1.25–1.51)	1.53 ± 0.07 (1.40–1.66)	0.15	0.058
E/È l	5.99 ± 0.25 (5.50–6.48)	5.64 ± 0.25 (5.15–6.13)	−0.35	0.064
E/È m	7.94 ± 0.25 (7.46–8.42)	7.23 ± 0.25 (6.75–7.71)	−0.71	**0** **.** 008*
TAPSE (mm)	25.84 ± 0.71 (24.50–27.22)	26.25 ± 0.71 (24.86–27.65)	0.41	0.642
LV GLS (%)	−20.94 ± 0.18 (−21.30 to −20.58)	−20.64 ± 0.19 (−21.01 to −20.27)	−0.30	0.078
LA strain reservoir (%)	44.48 ± 1.72 (41.11–47.86)	44.16 ± 1.75 (40.74–47.59)	−0.32	0.849
LA strain conduit (%)	−32.07 ± 1.79 (−35.58 to −28.56)	−31.63 ± 1.82 (−35.19 to −28.06)	−0.44	0.797
LA strain contraction (%)	−12.03 ± 0.58 (−13.17 to −10.89)	−12.51 ± 0.59 (−13.67 to −11.36)	0.48	0.332
RV GLS (%)	−22.04 ± 0.42 (−22.87 to −21.22)	−22.11 ± 0.42 (−22.94 to −21.28)	0.07	0.836
RV FWS (%)	−26.76 ± 0.99 (−28.69 to −24.82)	−24.93 ± 1.00 (−26.89 to −22.98)	−1.83	0.173

SE, Standard Error; LVEF, Left ventricular ejection fraction; LVEDV, Left ventricular end-diastolic volume; LVESV, Left ventricular end-systolic volume; LVIDd/s, Left ventricular internal diameter in diastole/systole; IVSd/s, Interventricular septal thickness in diastole/systole; LVPWd/s, Left ventricular posterior wall thickness in diastole/systole; SV, Stroke volume; E/A ratio; E/È l, E/È ratio lateral; E/È m, E/È ratio medial; TAPSE, Tricuspid annular plane systolic excursion; LV GLS, Left ventricular global longitudinal strain; LA, Left atrium; RV GLS, Right ventricular global longitudinal strain; RV FWS, Right ventricular free wall strain. Significant results are presented as follows: * ≤ 0.05.

At T0, LV GLS showed no significant associations with diastolic function parameters or exercise performance. There was a trend toward an association between LV GLS and the E/A ratio (*r* = –0.26, *p* = 0.064), whereas LV GLS was not associated with E/È lateral or medial (*r* = 0.13, *p* > 0.362) or with relative VO_₂peak_ (*r* = –0.13, *p* = 0.378). Right ventricular function remained unchanged over time, with no significant differences in TAPSE, RV GLS, or RV FWS (all *p* > 0.05). Overall, no adverse cardiac remodeling or deterioration in systolic function was observed.

Higher skeletal muscle mass was associated with slightly less negative LV GLS values in the univariable model (*b* = 0.236, *p* = 0.023), although the explained variance was low (adjusted R² = 0.045). In the multivariable model, skeletal muscle mass remained independent predictor of LV GLS (*b* = 0.414, *p* = 0.030), whereas age (*b* = 0.131, *p* = 0.207) and sex (*b* = 0.188, *p* = 0.314) were not significant predictors.

### 3.3 Cardiopulmonary performance and pulmonary function

**Figure 1:**
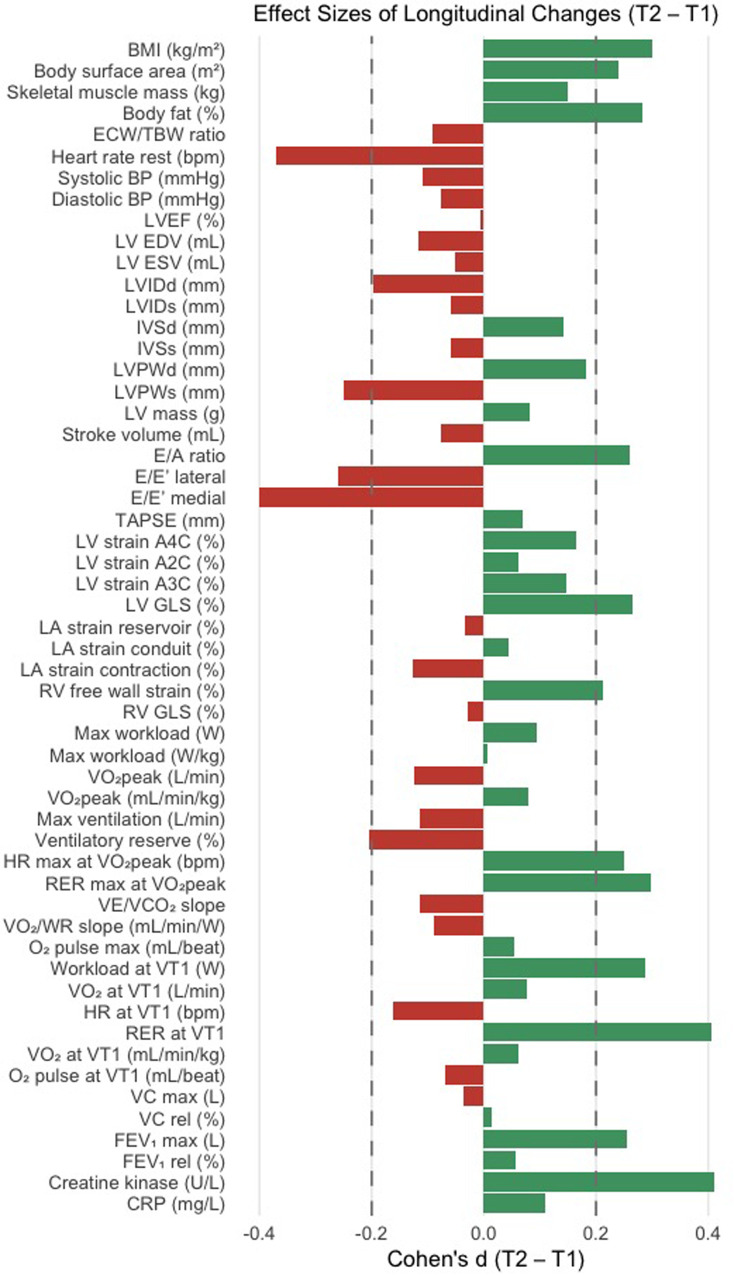
Effect sizes (Cohen`s d) of longitudinal changes (T1–T0) across clinical, echocardiographic, cardiopulmonary and pulmonary function parameters. BMI, Body Mass Index; ECW/TBW, Extracellular Water/Total Body Water Ratio; BP, Blood Pressure; LVEF, Left ventricular ejection fraction; LV EDV, Left ventricular end-diastolic volume; LV ESV, Left ventricular end-systolic volume; LVIDd/s, Left ventricular internal diameter in diastole/systole; IVSd/s, Interventricular septal thickness in diastole/systole; LVPWd/s, Left ventricular posterior wall thickness in diastole/systole; E/A ratio; E/È lateral, E/È ratio lateral; E/È medial, E/È ratio medial; TAPSE, Tricuspid annular plane systolic excursion; A4C, Four-chamber view; A2C, Two-chamber view; A3C, Three-chamber view; LV GLS, Left ventricular global longitudinal strain; LA, Left atrium; RV, Right ventricular; VO_2peak_, Peak respiratory oxygen uptake; HR, Heart Rate; RER, Peak respiratory exchange rate; VE/VCO_2_ slope, Ventilatory equivalent of carbon dioxide slope; VO_2_/WR slope, Oxygen uptake/work rate slope; O_2_pulse Oxygen Pulse, VT1, First Ventilatory Threshold; VC, Vital capacity; FEV1, Forced Expiratory Volume in 1 s; CRP, C-reactive protein.

Maximal exercise performance remained unchanged between T0 and T1 (Table 3). Maximum workload and workload normalized to body weight did not change significantly. VO_2peak_ in absolute terms showed no significant longitudinal difference, and relative VO_₂peak_ remained unchanged as well. Maximum ventilation, ventilatory reserve, peak oxygen pulse, VE/VCO_2_ slope, and VO_2_/WR slope demonstrated no significant changes over time. A small but statistically significant increase was observed in peak RER (T0: 1.22 ± 0.01 vs. T1: 1.25 ± 0.01 *p* = 0.045, Cohens`s d = 0.30). Maximum HR showed a trend toward higher values at T1 (T0: 167.5 ± 2.71 vs. T1: 172.5 ± 2.91, *p* = 0.092, Cohen`s d = 0.25). Pulmonary function parameters remained stable; including vital capacity (*p* = 0.787), relative VC (*p* = 0.914), FEV1 (*p* = 0.076), and relative FEV1 (*p* = 0.678) (Figure 1).

**Table 3:** Cardiopulmonary performance parameters at study time T0 and T1.

Variable	Mean ± SE T0 (95% CI)	Mean ± SE T1 (95% CI)	Change T1–T0	*p*-value
Maximum Workload (Watt)	270.1 ± 12.36 (245.9–294.3)	274.2 ± 12.39 (249.9–298.5)	+4.10	0.518
Maximum Workload/Body Weight (Watt/kg)	3.72 ± 0.16 (3.40–4.03)	3.72 ± 0.16 (3.40–4.03)	0.00	0.979
VO_2peak_ (L/min)	3.13 ± 0.37 (2.41–3.84)	2.69 ± 0.37 (1.97–3.41)	−0.44	0.382
Relative VO_2peak_ (mL/min/kg)	36.73 ± 1.56 (33.67–39.78)	37.31 ± 1.56 (34.25–40.38)	+0.58	0.174
Maximum Ventilation (L/min)	118.2 ± 5.92 (106.6–129.8)	114.2 ± 5.92 (102.6–125.8)	−4.00	0.463
Ventilatory Reserve (%)	83.42 ± 2.94 (77.65–89.19)	78.88 ± 2.94 (73.11–84.64)	−4.54	0.183
Maximum HR (bpm)	167.5 ± 2.71 (162.2–172.8)	172.5 ± 2.91 (166.8–178.2)	+5.00	0.092
RER	1.22 ± 0.01 (1.19–1.24)	1.25 ± 0.01 (1.22–1.27)	+0.03	**0** **.** 045*
VE/VCO_2_ slope	25.41 ± 0.49 (24.45–26.38)	24.90 ± 0.53 (23.87–25.93)	−0.51	0.398
VO_2_/WR slope	8.26 ± 0.21 (7.85–8.68)	8.05 ± 0.23 (7.60–8.49)	−0.21	0.409
Peak Oxygen Pulse (mL/beat)	15.42 ± 1.04 (13.38–17.46)	15.60 ± 1.11 (13.42–17.78)	+0.18	0.880
Vital Capacity (L)	4.80 ± 0.15 (4.51–5.10)	4.79 ± 0.15 (4.49–5.08)	−0.01	0.787
Relative Vital Capacity (%)	101.90 ± 1.89 (98.20–105.60)	102.00 ± 1.89 (98.31–105.70)	+0.01	0.914
FEV1 (L)	3.84 ± 0.12 (3.60–4.08)	3.93 ± 0.12 (3.69–4.17)	+0.09	0.076
Relative FEV1 (%)	99.33 ± 2.34 (94.75–103.90)	100.26 ± 2.35 (95.65–104.90)	+0.93	0.678

SE, Standard Error; VO_2peak_, Peak respiratory oxygen uptake; HR, Heart Rate; RER, Peak respiratory exchange rate; VE/VCO_2_ slope, Ventilatory equivalent of carbon dioxide slope; VO_2_/WR slope, Oxygen uptake/work rate slope; FEV1, Forced Expiratory Volume in 1 s. Significant results are presented as follows: *≤0.05.

### 3.4 Predictors of follow-up performance

In the multivariable linear regression including baseline VO_₂peak_, sex, and sport type, baseline VO_₂peak_ was the only independent predictor of VO_₂peak_ at T1 (*b* = 0.92, 95% CI 0.68–1.15, *p* < 0.001). Sex and sport category were no significant predictors. Resting myocardial deformation did not predict subsequent performance, as LV GLS at T0 was not associated with VO_₂peak_ at T1 (*b* = –0.56, *p* = 0.69; R² = 0.003). The final model explained 63.7% of the variance in follow-up VO_₂peak_.

## 4 Discussion

In this longitudinal cohort of elite and recreational athletes recovering from mild SARS-CoV-2 infection, cardiac structure, myocardial deformation, and cardiopulmonary performance remained stable over a 12-month period. No deterioration of LV systolic function was observed, as reflected by unchanged LVEF, LV volumes, and LV GLS. These findings are consistent with previous reports demonstrating preserved systolic function in athletes without clinical evidence of myocarditis or severe systemic involvement (20–22).

Diastolic function likewise remained stable. The small but statistically significant reduction in E/È medial suggested a slight decrease in estimated filling pressures, although the absolute change was minimal and likely not clinically relevant. Other diastolic indices demonstrated only nonsignificant trends, supporting an overall interpretation of preserved diastolic mechanics. These results align with our prior work, where recently infected athletes showed mildly altered E/A ratio and E/È lateral (14), and with findings in Post-COVID patients exhibiting modest E/A reduction despite values remaining within normal limits (15). Across studies, these subtle diastolic alterations appear transient, physiologic, and not clinically consequential.

At baseline, LV GLS showed no significant associations with diastolic parameters or exercise capacity. The weak, nonsignificant trend toward an association with the E/A ratio likely reflects normal physiologic variation among trained individuals rather than impaired diastolic function. Likewise, the absence of associations between E/È medial and LA strain components supports intact atrial-ventricular coupling. Right ventricular parameters (TAPSE, RV GLS/FWS) also remained unchanged, paralleling findings from elite athletes (21), healthy young adults (34) and PCS cohorts (15). Collectively, these data indicate that mild SARS-CoV-2 infection does not induce adverse cardiac remodeling or progressive myocardial impairment in otherwise healthy, physically active individuals.

A notable secondary finding was that skeletal muscle mass independently predicted LV GLS. Although the explained variance was modest, the association likely reflects physiological coupling between lean mass, blood and hemoglobin volume, and preload—well-established components of the athletès heart. Athletes with higher skeletal muscle mass typically exhibit increased circulating blood volume and hemoglobin mass (35), which may influence longitudinal strain due to its load dependency. While this observation is physiologically plausible, the effect size was small and warrants confirmation in larger athlete cohorts.

Beyond myocardial function, cardiopulmonary performance remained remarkably stable. Neither maximal workload nor relative or absolute VO_₂peak_ showed significant longitudinal change, consistent with previous studies demonstrating preserved or rapidly normalized aerobic capacity and ventilatory efficiency in athletes following mild SARS-CoV-2 infection (36–38). Measures of ventilatory efficiency—including VE/VCO_2_ slope, ventilatory reserve, oxygen pulse, and VO_2_/WR slope—as well as pulmonary function parameters likewise remained unchanged, aligning with reports of preserved ventilatory mechanics and gas-exchange efficiency in Post-COVID athletes without cardiac involvement (20, 36–38). Although some studies have reported abnormal spirometry and reduced breathing reserve in athletes with persistent symptoms early after infection (37), our asymptomatic cohort and the 12-month follow-up interval suggest full physiologic recovery.

The small increase in peak RER and the nonsignificant trend toward higher maximal heart rate may superficially suggest subtle changes in exertional tolerance. However, the negligible effect sizes, absence of concomitant reductions in VO_2peak_, and stable ventilatory efficiency strongly support physiological or test-retest variability rather than true impairment. This interpretation is consistent with evidence showing that day-to-day fluctuations in heart rate and metabolic responses do not reflect meaningful changes in aerobic capacity (39).

In contrast, studies in non-athletic or clinically vulnerable populations consistently report persistent reductions in VO_₂peak_, ventilatory inefficiency, and exertional dyspnea months after infection—even following mild or moderate disease (40–42). Proposed mechanisms include deconditioning, autonomic dysregulation, respiratory muscle weakness, or residual pulmonary involvement (43–45). The stark contrast to our findings underscores the well-documented protective effects of regular endurance and mixed training, including increased blood and hemoglobin volume (35), enhanced stroke volume reserve and cardiac compliance (46), improved mitochondrial density and oxidative function (47, 48), and more efficient ventilatory control (49). Accordingly, our results highlight not only the benign trajectory after mild COVID-19 in athletes but also the potential preventive role of habitual physical activity in mitigating cardiopulmonary consequences of viral illness—independent of sport discipline.

Baseline VO_₂peak_ emerged as the strongest independent predictor of follow-up VO_₂peak_, explaining nearly two thirds of its variance. Neither sex, sport discipline, nor resting echocardiographic parameters, including LV GLS, RV deformation, or diastolic indices, contributed additional explanatory value. Consistent with our previous findings in athletes recovering from COVID-19 (18), neither cross-sectional nor longitudinal associations were observed between myocardial deformation (LV GLS), filling pressures (E/È), and aerobic capacity (VO_₂peak_). These results further support that resting cardiac function does not limit performance recovery after mild SARS-CoV-2 infection. Instead, baseline fitness and peripheral adaptations appear to be the principal determinants.

Taken together, our findings provide reassuring evidence that in athletes who experience mild COVID-19, both cardiac health and exercise performance remain intact, with no indication of persistent myocardial impairment or diminished cardiopulmonary reserve. Thus, athletes who have recovered from a mild SARS-CoV-2 infection and are clinically asymptomatic may safely resume training and competition after standard medical clearance. These results underscore the favorable cardiovascular prognosis and highlight the protective role of regular athletic training and high cardiorespiratory fitness.

### 4.1 Strengths and limitations

This study has several notable strengths. It integrates advanced echocardiographic deformation imaging, comprehensive CPET, and body composition analysis within a prospective longitudinal design. All assessments were conducted using standardized protocols by experienced investigators, minimizing interobserver variability. The 12-month follow-up period provides rare long-term data on cardiovascular recovery in athletes, and the inclusion of participants from multiple sport disciplines enhances generalizability within trained populations.

Several limitations should also be acknowledged. First, the sample size was modest, which may have reduced statistical power to detect small associations, particularly in multivariable analyses. Second, only two assessment time points are available, limiting the ability to characterize individual recovery trajectories. Third, strain measurements relied on a single vendor-specific platform, which may restrict generalizability and is subject to test-retest and load dependent variability. Fourth, CPET was performed exclusively on a cycle ergometer and may underestimate maximal performance in non-cycling athletes. Fifth, training load and detraining effects between T0 and T1 were not systematically recorded and may have contributed to changes in VO_₂peak_ or body composition. Additionally, the cohort included athletes from different sport disciplines and training levels, including both elite and recreational athletes, which may have introduced heterogeneity in cardiac adaptations and performance parameters. Sex-specific analyses were not performed due to limited subgroup sizes and should be considered in larger future cohorts. Finally, the findings apply only to athletes with mild SARS-CoV-2 infection and cannot be extrapolated to those with more severe disease or persistent symptoms.

## 5 Conclusion

In athletes recovering from mild SARS-CoV-2 infection, cardiac structure, myocardial deformation, and cardiopulmonary performance remained stable over 12 months, with no evidence of adverse remodeling or functional impairment. These findings suggest a stable a 1-year cardiopulmonary outcome and support the safety of return to sport after mild COVID-19.

## Data Availability

The original contributions presented in the study are included in the article/Supplementary Material, further inquiries can be directed to the corresponding author/s.
